# Prenatal exposure to binge pattern of alcohol consumption: mental health and learning outcomes at age 11

**DOI:** 10.1007/s00787-014-0599-7

**Published:** 2014-09-11

**Authors:** Kapil Sayal, Jon Heron, Elizabeth Draper, Rosa Alati, Sarah J. Lewis, Robert Fraser, Margaret Barrow, Jean Golding, Alan Emond, George Davey Smith, Ron Gray

**Affiliations:** 1 Division of Psychiatry and Applied Psychology, School of Medicine, University of Nottingham, E Floor, South Block, Queen’s Medical Centre, Nottingham, NG7 2UH UK; 2 School of Social and Community Medicine, University of Bristol, Bristol, UK; 3 Department of Health Sciences, University of Leicester, Leicester, UK; 4 School of Population Health, University of Queensland, Herston, Australia; 5 Reproductive and Developmental Medicine, University of Sheffield, Sheffield, UK; 6 Department of Clinical Genetics, University Hospitals of Leicester NHS Trust, Leicester, UK; 7 National Perinatal Epidemiology Unit, University of Oxford, Oxford, UK; 8 CANDAL (Centre for ADHD and Neuro-developmental Disorders across the Lifespan), Institute of Mental Health, Nottingham, UK

**Keywords:** Prenatal alcohol exposure, Fetal alcohol spectrum disorder, Academic achievement, Mental health problems, Hyperactivity

## Abstract

The objective of the study is to investigate whether episodic binge pattern of alcohol consumption during pregnancy is independently associated with child mental health and academic outcomes. Using data from the prospective, population-based Avon Longitudinal Study of Parents and Children (ALSPAC), we investigated the associations between binge patterns of alcohol consumption during pregnancy (≥4 drinks per day) and child mental health [as rated by both parent (*n* = 4,610) and teacher (*n* = 4,274)] and academic outcomes [based on examination results (*n* = 6,939)] at age 11 years. After adjusting for prenatal and postnatal risk factors, binge pattern of alcohol consumption (≥4 drinks in a day on at least one occasion) during pregnancy was associated with higher levels of mental health problems (especially hyperactivity/inattention) in girls at age 11 years, according to parental report. After disentangling binge-pattern and daily drinking, binge-pattern drinking was independently associated with teacher-rated hyperactivity/inattention and lower academic scores in both genders. Episodic drinking involving ≥4 drinks per day during pregnancy may increase risk for child mental health problems and lower academic attainment even if daily average levels of alcohol consumption are low. Episodic binge pattern of drinking appears to be a risk factor for these outcomes, especially hyperactivity and inattention problems, in the absence of daily drinking.

**Electronic supplementary material:**

The online version of this article (doi:10.1007/s00787-014-0599-7) contains supplementary material, which is available to authorized users.

Alcohol consumption during pregnancy can lead to fetal alcohol spectrum disorders amongst exposed children; outcomes include adverse physical, developmental and behavioural consequences [1]. Fetal exposure to high peak levels of maternal blood alcohol may pose particular risks for childhood behavioural and learning problems. Following a systematic review of the literature, recommendations have been made for investigation of the risk to offspring through episodic heavier drinking such as binge patterns of drinking in women whose average levels of alcohol consumption during pregnancy are low to moderate [2]. A number of studies have investigated the impact of prenatal alcohol exposure on childhood mental health, learning and development [3–15]. However, only some of these studies have found associations between binge patterns of drinking specifically and childhood mental health or learning problems [3–10, 15]. Discrepancies in studies’ findings might reflect methodological factors such as confounding, choice of sample, source of informant for outcome data or ascertainment of exposure information postnatally [2, 8, 13, 15]. Furthermore, there are differences between studies in the quantification used to define the ‘binge’ pattern; for women, the National Institute on Alcohol Abuse and Alcoholism (NIAAA) National Advisory Council and the Centers for Disease Control and Prevention define the consumption of ≥4 drinks on a single occasion as binge drinking [17]. In terms of outcomes, some effects, particularly academic impairments, may only become apparent as children get older [2, 15, 16]. We aim to address some of these methodological issues using data from a prospective population-based study and adjusting for a range of prenatal and postnatal risk factors to assess outcomes at age 11 years. In a previous analysis of this population-based cohort, we found that a binge pattern of drinking during pregnancy was associated with adverse parent-rated mental health outcomes at ages 4 and 7 years, particularly in girls [18]. In the present study, we extend this work to a broader range of outcomes (parent-rated mental health, teacher-rated mental health, and academic achievement) measured at the age of 11 years. We aim to investigate: (1) the impact of episodic binge-pattern drinking on mental health as rated by both parents and teachers and on academic achievement and (2) whether risk occurs independently of regular (daily) drinking.

## Method

### Sample

The Avon Longitudinal Study of Parents and Children (ALSPAC) is a prospective population-based study in England [19]. All pregnant women resident in the Avon area with an expected delivery date between April 1991 and December 1992 were invited to participate. Around 85 % of all eligible women participated, providing a cohort of 14,541 pregnancies. ALSPAC participants were broadly representative of the local population of mothers with infants and comparable against national census data although they were slightly more likely to be Caucasian, married or cohabiting, home owner-occupiers, and have a car in the household (further details at http://www.alspac.bris.ac.uk and http://www.bristol.ac.uk/alspac/researchers/resources-available/cohort/represent/). Detailed information on the cohort (mothers and children) has been obtained through questionnaires completed at regular intervals during the pregnancy and since the birth. Please note that the study website contains details of all the data that are available through a fully searchable data dictionary (http://www.bris.ac.uk/alspac/researchers/data-access/data-dictionary/). Ethical approval for the study was obtained from the ALSPAC Ethics and Law Committee and the Local Research Ethics Committees.

To minimise confounding and clustering effects, the sample for analysis (see online Figure-Flow Chart) was restricted to women of white-European ethnicity (reflecting variations in alcohol dehydrogenase allele frequency across different populations [20]) and to children from singleton births (*n* = 13,171). Using postal questionnaires, information on alcohol consumption at 18 weeks gestation was gathered on 93 % (*n* = 12,257) of their mothers. Of these respondents, 65 % (*n* = 7,965) provided further information on alcohol consumption at 32 weeks gestation. This discrepancy reflects 3,378 (28 %) mothers who completed the initial version of the 32-week questionnaire where these questions were not asked and non-response in 914 (7 %) mothers. As previously shown, there was no sampling bias in the mothers not asked these questions [18]. However, maternal correlates of not providing this information when asked included the consumption of ≥4 drinks in a day on at least one occasion between 14 and 18 weeks gestation, smoking and use of cannabis in the early part of the second trimester, lower maternal age and level of education, depression, being unmarried, and living in rented housing.

### Measures

#### Exposure variables

Alcohol consumption during pregnancy was measured in the following ways:

1. Pattern of drinking reflecting episodic heavy drinking (primary exposure variable)—at both 18 and 32 weeks gestation, the mother was asked the number of days in the previous 4 weeks (i.e. reflecting 14–18 and 28–32 weeks gestation) on which she had consumed the equivalent of at least 4 units of alcohol. Examples were provided to confirm that one drink was equivalent to 1 unit (8 g) of alcohol. For analyses, this primary exposure variable reflected the consumption of ≥4 drinks in a day (binge-pattern drinking) on at least one occasion versus none.
2. Frequency and quantity of drinking—at 18 weeks, the mother was asked the frequency and quantity of her drinking during the previous 2 weeks or around the time she first felt the baby move. Response categories provided were never, <1 glass per week, ≥1 glass per week, 1–2 glasses a day, 3–9 glasses a day or ≥10 glasses a day. At 32 weeks, she was asked the amount she usually drank in a day at that time. For analyses, daily drinking was defined as an average of at least one drink a day at either time point.

Information was also obtained on alcohol consumption during two other time periods:

1. First trimester—information about drinking in the first trimester was also collected at 18 weeks gestation using the same frequency/quantity response categories as described above. Based on our previous work, for analyses, the groups consuming ≥1 glasses per week during the first trimester were combined [21].
2. When the child was aged 5 years, information was collected on the mother’s pattern of drinking during the past month. This was the last time point before the age of 11 when information was collected on maternal drinking patterns. As above, the consumption of ≥4 units of alcohol on any day was used as an index of postnatal binge pattern of drinking.

#### Outcome variables

1. Child mental health outcomes were measured using both the parent- and teacher-completed Strengths and Difficulties Questionnaire (SDQ) at the age of 11 years [22]. These were administered by post and followed by reminder questionnaires if there was no response. The SDQ is a widely used dimensional measure of childhood mental health and has been validated in a large, nationally representative, community sample [23]. It includes four sub-scales relating to emotional problems, conduct problems, hyperactivity/inattention, and peer relationships and higher scores (scale of 0–10) indicate greater levels of severity. These are summed to provide a total problems score (0–40). Our analyses focus on the total problems score as well as the two behavioural problem sub-scales (conduct problems and hyperactivity/inattention).
2. Academic outcomes were assessed using standardised, age-adjusted total scores from results on the Key Stage 2 (KS2) examinations taken in the final year at primary school (at ages 10–11 years). These scores provide an objective real world measure of academic performance and were obtained by data linkage with the National Pupil Database. In England, the national curriculum at KS2 relates to the school years 3–6, covering ages 7–11. Formal assessments involving examinations in English, Mathematics and Science take place at the end of this KS2 period. Further details are available at http://curriculum.qcda.gov.uk/key-stages-1-and-2/index.aspx.

#### Confounding variables

A range of potential confounding factors (selected due to being likely risk factors for the outcome in addition to being correlates of the exposure) that were measured in ALSPAC were included in the analyses. Based on our earlier work [18], the following prenatal maternal and socio-demographic variables obtained during the pregnancy were categorised for analysis: maternal age (≤20, 21–34, or ≥35 years); parity (none or ≥1); highest level of maternal education (based on the UK examination system- <‘O’ levels, ‘O’ levels, ‘A’ levels, degree); the number of times per day the mother smoked during the second trimester (response categories were 0, 1–4, 5–9, 10–14, 15–19, 20–24, 25–29, and 30+); and use of cannabis and other illicit drugs during the pregnancy, home ownership and whether currently married (all dichotomized to yes/no). Maternal mental health was measured at 18 weeks gestation and when the child was 11 years old using the Edinburgh Postnatal Depression Scale (EPDS)—this measure has been validated outside the postnatal period [24]. As scores of >12 are highly associated with a diagnosis of a depressive disorder, this cut off was used to indicate the presence of depression [25]. Analyses also adjusted for child factors including gestational age (≤36 or ≥37 weeks), birth weight and gender. As parental and child educational attainment are strongly associated, analyses for KS2 outcomes were further adjusted for highest level of paternal education (as reported by the mother and categorised similarly).

### Data analysis

The main analyses consisted of linear regression models examining the association between the consumption of ≥4 drinks in a day on at least one occasion (yes/no; main exposure) at either measured time point during pregnancy and child SDQ and KS2 outcomes at age 11. The sample was restricted to the 7,965 children whose mothers provided information on alcohol use at both 18 and 32 weeks gestation. Regression estimates were adjusted for the potential confounding effect of the prenatal variables as well as child factors listed above using multivariable regression modelling. Based on our earlier work [18], the following additional steps were carried out:

1. We identified the relationships between the main exposure (≥4 drinks per day) and the prenatal maternal and socio-demographic and child variables outlined above (Table 1).

**Table 1:** Correlates of consuming ≥4 drinks in a day during pregnancy

	<4 drinks a day *n* = 6044	≥4 drinks a day *n* = 1921	*χ* ^2^ (1 d.f.)	*p*
Prenatal factors				
Maternal age (≥35 years)	10 %	11 %	1.56	0.211
Any smoking	15 %	30 %	209.44	<0.001
Cannabis use	1 %	4 %	62.41	<0.001
Other illicit drug use	0.3 %	0.8 %	7.20	0.007
Parity (≥1)	54 %	61 %	26.34	<0.001
Highest maternal education (O level or above)	73 %	63 %	77.18	<0.001
Own home	79 %	70 %	65.28	<0.001
Married	81 %	71 %	81.20	<0.001
Maternal depression	11 %	18 %	60.54	<0.001
Child factors				
Gestational age (≤36 weeks)	4 %	4 %	0.06	0.802
Gender (male)	51 %	52 %	0.78	0.377
Birth weight (kg)	3.44 (0.52)	3.44 (0.54)	*t* = 0.11^a^	0.915

% or mean(s.d.)

^a^ *t* test, *t* = 0.11
2. To check for possible selection bias we assessed whether the availability of outcome measures (SDQ and KS2 data) was associated with the exposure and other confounding variables (listed above). These assessments were made using *χ* ^2^ or *t* tests as appropriate.
3. To assess for possible risk associated with background alcohol consumption at other times outside of pregnancy as opposed to the risk from exposure during pregnancy (intra-uterine exposure), we examined the univariable relationships between postnatal binge pattern of drinking (when the child was aged 5 years) and outcome measures.
4. We formally tested for possible gender interaction within the unadjusted models and, where appropriate, regression analyses were repeated separately by gender.
5. Three sets of sensitivity analyses were carried out for the parent-rated SDQ outcomes. First, given our earlier findings, the multivariable linear regression analyses were repeated after adjusting for first trimester alcohol consumption [21]. Second, given the possibility that the child’s gestational age and birth weight might be on the causal pathway between prenatal alcohol exposure and neuro-developmental problems, the multivariable linear regression analyses were repeated after omitting these variables from the model. Third, the multivariable linear regression analyses were repeated after including maternal binge pattern of drinking at 5 years and EPDS at 11 years in the model. This analysis adjusted for the effects of key risk factors in the postnatal environment.
6. Finally, to examine whether episodes of heavier drinking may carry risk of later adverse outcomes in mothers who do not drink daily during pregnancy, the primary analyses were repeated using a 4-level variable of drinking in pregnancy separating out patterns (≥4 drinks in a day) and frequency (daily drinking involving an average of at least one drink per day) of drinking. We compared, in turn, three sub-groups of mothers (those who drank daily but not ≥4, ≥4 but not daily, ≥4 and daily) against the baseline group who had neither consumed ≥4 drinks in a day nor drank daily during the pregnancy. The analyses for KS2 outcomes were further adjusted for highest level of paternal education.

## Results

A quarter (24 %; 1,921/7,965) of mothers reported consuming ≥4 drinks in a day on at least one occasion during pregnancy. Over half [56 % (725/1,305) and 57 % (790/1,390) at each time point] of these mothers had consumed ≥4 drinks on only 1–2 days in the previous month. The majority (59 %; 774/1,305) of mothers who reported at least one occasion of consuming ≥4 drinks between 14 and 18 weeks also reported this pattern between 28 and 32 weeks gestation. Table 1 shows that maternal correlates of consuming ≥4 drinks included higher parity, smoking, use of cannabis and other illicit drugs, depression, being unmarried, rented tenure, and lower level of education.

Parent-completed SDQs were available on 4,610 (58 %) children. Mothers who reported consuming ≥4 drinks were less likely than other mothers to provide SDQs (52 vs. 60 %; *χ* ^2^ = 40.08, *p* < 0.001). Other maternal correlates of non-response included younger age, higher parity, smoking and use of cannabis, depression, being unmarried, rented tenure, and lower level of education (Online Supplementary Table 1). Teacher-completed SDQs were available on 4,274 (54 %) and KS2 data on 6,939 (87 %) children. Their availability (Online Supplementary Tables 2 and 3) was not associated with drinking status, but other correlates of non-availability included use of cannabis, being unmarried and rented tenure (both SDQ and KS2), and lower parity and higher level of maternal education (KS2 only).

Postnatal binge pattern of alcohol consumption (child aged 5 years) was not adversely associated with SDQ or KS2 outcome measures.

### Relationships between alcohol consumption during pregnancy and outcomes

There was evidence of an interaction between child gender and alcohol consumption in relation to parent-rated SDQ scores (Table 2). In girls, the effect sizes of the unadjusted differences were up to 0.2 of a standard deviation. In adjusted models, the consumption of ≥4 drinks was associated with hyperactivity/inattention, conduct and total problems in girls. These patterns of associations persisted, with some attenuation, in all three sensitivity analyses (i.e. excluding gestational age and birth weight from the model and after adjusting for first trimester alcohol consumption and postnatal variables). Although there were similar associations involving teacher-rated SDQ and KS2 scores in the univariable analyses, these did not persist after adjusting for confounders (Table 3).

**Table 2:** Relationships between binge pattern of drinking (yes/no) and mean differences (regression coefficients) in parent-rated SDQ

Outcome measures (score range)	Unadjusted mean differences (95 % C.I.)	*p*	Adjusted^b^ mean differences (95 % C.I.)	*p*	*p* for gender interaction^a^
Whole sample					
Conduct problems (0–10)	0.16 (0.06,0.26)	0.002	0.05 (−0.06,0.15)	0.406	0.105
Hyperactivity/inattention (0–10)	0.27 (0.12,0.43)	0.001	0.11 (−0.05,0.27)	0.178	0.082
Total problems (0–40)	0.68 (0.33,1.03)	<0.001	0.30 (−0.07,0.67)	0.109	0.022
Boys					
Conduct problems (0–10)	0.08 (−0.07,0.22)	0.314	−0.06 (−0.21,0.10)	0.460	
Hyperactivity/inattention (0–10)	0.14 (−0.09,0.36)	0.245	−0.02 (−0.26,0.23)	0.889	
Total problems (0–40)	0.27 (−0.23,0.78)	0.288	−0.16 (−0.69,0.37)	0.565	
Girls					
Conduct problems (0–10)	0.24 (0.11,0.37)	<0.001	0.16 (0.01,0.31)	0.034	
Hyperactivity/inattention (0–10)	0.40 (0.20,0.60)	<0.001	0.25 (0.04,0.47)	0.022	
Total problems (0–40)	1.08 (0.61,1.56)	<0.001	0.80 (0.29,1.31)	0.002	

^a^In unadjusted model

^b^Adjusted for (multivariable analysis *n* = 4,183): maternal age, parity, highest level of maternal education, daily frequency of smoking during the second trimester, use of cannabis and/or other illicit drugs in pregnancy, home ownership, whether currently married, high scores (>12) on the Edinburgh Postnatal Depression Scale, and child gestational age, birth weight and gender

**Table 3:** Relationships between binge pattern of drinking (yes/no) and mean differences (regression coefficients) in teacher-rated SDQ and Key stage 2

Outcome measures (score range)	Unadjusted mean differences (95 % C.I.)	*p*	Adjusted^b^ mean differences (95 % C.I.)	*p*	*p* for gender interaction^a^
Conduct problems (0–10)	0.17 (0.06,0.28)	0.003	−0.03 (−0.15,0.08)	0.546	0.151
Hyperactivity/inattention (0–10)	0.36 (0.17,0.54)	<0.001	0.11 (−0.08,0.29)	0.255	0.860
Total problems (0–40)	0.43 (0.02,0.83)	0.039	−0.20 (−0.61,0.21)	0.332	0.652
Key stage 2	−1.82 (−1.31,−2.32)	<0.001	−0.32 (−0.82,0.18)	0.212	0.275

^a^In unadjusted model

^b^Adjusted for (multivariable analyses: teacher-rated SDQ *n* = 3,726; Key stage 2 *n* = 6,035): maternal age, parity, highest level of maternal education, daily frequency of smoking during the second trimester, use of cannabis and/or other illicit drugs in pregnancy, home ownership, whether currently married, high scores (>12) on the Edinburgh Postnatal Depression Scale, and child gestational age, birth weight and gender

At either time point, 8 % (522/6,272) of mothers reported regular daily drinking, i.e. at least one drink a day in the preceding month (between 14 and 18 or 28 and 32 weeks gestation). Overall, 19 % of mothers reported binge-pattern drinking, but not daily drinking whereas 5 % reported both episodic binge-pattern drinking and daily drinking. The relationships between patterns and frequency/quantity of drinking during pregnancy and outcomes are shown in Table 4 (the reference category includes both non-drinkers and light drinkers (i.e. participants who did not drink daily or have any episodes of binge-pattern drinking)). After adjustment for confounders, an episodic pattern of consuming ≥4 drinks in a day in the absence of daily drinking was associated with more hyperactivity/inattention and total problems in girls, according to the parent. Furthermore, across both genders, there was an association with more teacher-rated hyperactivity/inattention problems. This episodic pattern of binge drinking was also associated with lower KS2 scores and this association persisted after adjusting further for paternal education (adjusted regression coefficient = −0.82, 95 % CI −0.16 to −1.47, *p* = 0.016). In contrast, daily (non-binge) drinking in the absence of binge pattern of drinking was not associated with KS2 scores after adjusting for paternal education (adjusted regression coefficient = 0.85, 95 % CI −0.46 to 2.16, *p* = 0.203).

**Table 4:** Relationships between binge drinking patterns and mean differences in SDQ scores in the presence or absence of daily drinking

Outcome measures (score range)	Unadjusted mean differences (95 % C.I.)	*p*	Adjusted mean differences (95 % C.I.)	*p*
Parent SDQ (boys)				
Conduct problems (0–10)				
Daily drinking but not 4+	−0.02 (−0.40,0.37)	0.927	0.03 (−0.36,0.41)	0.895
4+ but not daily drinking	0.20 (0.00,0.40)	0.049	0.03 (−0.17,0.23)	0.769
4+ and daily drinking	0.01 (−0.30,0.33)	0.933	−0.22 (−0.55,0.11)	0.185
Hyperactivity/inattention (0–10)				
Daily drinking but not 4+	0.04 (−0.55,0.63)	0.903	0.18 (−0.42,0.77)	0.561
4+ but not daily drinking	0.09 (−0.21,0.39)	0.542	−0.11 (−0.42,0.21)	0.507
4+ and daily drinking	0.16 (−0.32,0.64)	0.515	−0.21 (−0.72,0.31)	0.431
Total problems (0–40)				
Daily drinking but not 4+	−0.08 (−1.39,1.24)	0.910	0.27 (−1.03,1.57)	0.680
4+ but not daily drinking	0.43 (−0.24,1.10)	0.212	−0.09 (−0.78,0.59)	0.788
4+ and daily drinking	0.13 (−0.95,1.21)	0.816	−0.76 (−1.89,0.37)	0.187
Parent SDQ (girls)				
Conduct problems (0–10)				
Daily drinking but not 4+	−0.21 (−0.53,0.11)	0.199	−0.19 (−0.53,0.15)	0.266
4+ but not daily drinking	0.27 (0.09,0.45)	0.003	0.18 (−0.01,0.37)	0.067
4+ and daily drinking	0.16 (−0.14,0.46)	0.288	0.13 (−0.18,0.44)	0.399
Hyperactivity/inattention (0–10)				
Daily drinking but not 4+	0.03 (−0.44,0.50)	0.904	0.19 (−0.30,0.68)	0.453
4+ but not daily drinking	0.49 (0.23,0.75)	<0.001	0.30 (0.02,0.58)	0.035
4+ and daily drinking	0.41 (−0.02,0.84)	0.064	0.36 (−0.09,0.81)	0.119
Total problems (0–40)				
Daily drinking but not 4+	−0.52 (−1.64,0.59)	0.357	−0.30 (−1.47,0.87)	0.617
4+ but not daily drinking	1.24 (0.62,1.86)	<0.001	0.99 (0.32,1.65)	0.004
4+ and daily drinking	0.93 (−0.10,1.97)	0.076	0.78 (−0.30,1.85)	0.157
Teacher SDQ (whole sample)				
Conduct problems (0–10)				
Daily drinking but not 4+	−0.08 (−0.39,0.24)	0.633	0.06 (−0.25,0.36)	0.722
4+ but not daily drinking	0.19 (0.05,0.34)	0.009	0.02 (−0.13,0.16)	0.811
4+ and daily drinking	−0.02 (−0.24,0.21)	0.900	−0.14 (−0.36,0.09)	0.238
Hyperactivity/inattention (0–10)				
Daily drinking but not 4+	−0.29 (−0.82,0.24)	0.281	0.01 (−0.48,0.51)	0.956
4+ but not daily drinking	0.50 (0.25,0.74)	<0.001	0.28 (0.04,0.51)	0.024
4+ and daily drinking	0.13 (-0.25,0.52)	0.497	0.03 (-0.35,0.40)	0.891
Total problems (0–40)				
Daily drinking but not 4+	−0.50 (−1.68,0.67)	0.403	−0.03 (−1.15,1.09)	0.957
4+ but not daily drinking	0.70 (0.16,1.24)	0.010	0.16 (−0.38,0.70)	0.566
4+ and daily drinking	−0.25 (−1.10,0.60)	0.560	−0.53 (−1.37,0.31)	0.216
Key stage 2 (whole sample)				
Daily but not 4+	3.15 (1.73,4.58)	<0.001	1.37 (0.02,2.72)	0.047
4+ but not daily drinking	−2.37 (−1.71,−3.03)	<0.001	−0.81 (−0.16,−1.46)	0.014
4+ and daily drinking	−0.87 (−1.96,0.22)	0.116	0.10 (−0.95,1.15)	0.850

Baseline/Reference category: not drinking daily nor any episodic consumption of ≥4 drinks in a day

Daily but not 4+: drinking an average of ≥1 drink a day, but no episodic consumption of ≥4 drinks in a day

4+ but not daily drinking: episodic consumption of ≥4 drinks in a day amongst women who did not drink an average of ≥1 drink a day

4+ and daily drinking: episodic consumption of ≥4 drinks in a day amongst women who also drank an average of ≥1 drink a day

## Discussion

After adjusting for prenatal confounding factors and disentangling binge-pattern and daily drinking, our findings suggest that a binge pattern of alcohol consumption during pregnancy (≥4 drinks in a day) is independently associated with higher levels of hyperactivity and inattention problems and lower academic attainment at age 11 years (Table 4). This pattern of drinking appeared to be an independent risk factor for these outcomes amongst offspring of mothers who did not drink daily during pregnancy, but only on an episodic basis. As in our earlier work [18], parent ratings suggested a higher risk of mental health problems amongst girls exposed to this episodic binge pattern of drinking. However, when outcomes were assessed using teacher ratings and academic attainment scores, both genders appear to be at risk through exposure to episodic binge-pattern drinking in the absence of daily drinking. In contrast, moderate levels of alcohol consumption (averaging at least one drink a day) during pregnancy in the absence of binge pattern of drinking were not associated with adverse outcomes at age 11.

With regard to mental health outcomes, the strongest associations were with hyperactivity/inattention. This association was reflected across both parent and teacher ratings and was not contingent upon daily drinking during pregnancy. There are inconsistent findings about the association between prenatal alcohol exposure and childhood hyperactivity and inattention problems [18, 26, 27]. This might reflect methodological issues—although recommendations have been made for research that separates out risk from regular drinking versus episodic binge-pattern drinking [2], few studies have separated these out to specifically examine the association between binge-pattern drinking during pregnancy and hyperactivity and inattention problems [10, 18]. Some studies have conflated frequency (e.g. daily) and pattern (e.g. binge episodes) of drinking or have ascertained prenatal alcohol exposure status postnatally [8, 13, 15, 16, 28]. Some studies suggest the possibility that the mental health risk to children prenatally exposed to alcohol mainly reflects conduct and emotional problems [15, 16, 28, 29]. Although, for girls, we also found higher levels of parent-rated conduct problems, these findings did not extend to teacher ratings.

Several studies investigating cognitive outcomes following prenatal exposure to binge pattern of drinking have not found adverse effects on global intellectual functioning such as IQ scores [11–14, 18, 30]. However, specific associations have been found with lower verbal and non-verbal IQ, particularly if binge episodes are frequent [7, 9, 10]. Relatively few studies have focused on school-based learning or academic outcomes. The Seattle longitudinal study found that binge-pattern drinking (≥60 g alcohol per occasion) was associated with childhood learning problems that persisted over time [3, 4]. Specific problems with maths, reading and verbal memory have also been described [3, 6]. After adjustment for confounders, our findings have shown an adverse impact of occasional drinking episodes involving ≥4 drinks in a single day (involving ≥32 g of alcohol) on children’s academic attainment at age 11. These findings are consistent with animal studies which demonstrate adverse effects on learning, hyperactivity/inattention, and executive function through exposure to high peak alcohol levels [31, 33].

Although some animal studies have suggested a greater vulnerability to prenatal alcohol exposure amongst female offspring [33–35], it remains uncertain whether the parent SDQ findings reflect informant-related factors, whereby the parent (usually mother) has provided both the exposure and outcome information. However, the finding of higher vulnerability amongst girls is consistent with our previous follow-up of this cohort up until age 7 years and the association remained robust even after adjusting for postnatal environmental risk factors as well as first trimester exposure [18].

ALSPAC is well placed to answer the question of whether episodic binge-pattern drinking in pregnancy is associated with risk to offspring. Data were collected prospectively during pregnancy, the sample reflects a large population-based cohort and outcome data were obtained from a range of sources. Another advantage is that these exposure data were collected during 1991–1992 when attitudes to drinking in pregnancy were different in the UK and there was less stigma about reporting alcohol consumption whilst pregnant. The majority of mothers who reported consuming ≥4 drinks in a day at the first time point also reported this at the second, suggesting that this pattern of drinking might have persisted across the second and third trimesters. However, if under-reporting and drinking that takes place prior to pregnancy recognition are also taken into account, we may have under-estimated the level of exposure. The multivariable analyses adjusted for a wide range of factors that were potentially associated with the exposure and outcome, including paternal characteristics and postnatal factors such as maternal mental health and binge pattern of drinking. Furthermore, there was no adverse association between postnatal binge-pattern drinking (child aged 5 years) and outcomes, suggesting that risk related to intra-uterine exposure. In the analyses, we were also able to separate out the effects of frequency and quantity of drinking from binge-pattern drinking. This enabled us to achieve our aim of distinguishing risk related to occasional episodes of heavier drinking [36]. As the study utilised a population-based sample, few mothers (5 % of the sample) reported both daily drinking as well as episodic binge-pattern drinking which might have limited our ability to demonstrate an association involving this group. It is also possible that they spread their drinking over the course of the day when consuming ≥4 drinks and that their alcohol metabolising enzymes have been sufficiently induced because of their daily drinking pattern resulting in reduced fetal exposure to peak alcohol levels.

In terms of limitations, our ascertainment of the timing of the exposure meant that it was not possible to specify any particular risk period during the pregnancy. We did not have information on first trimester binge-pattern drinking. The differences in our findings compared to other studies may also reflect other methodological factors such as sample size, ascertainment of drinking behaviour by self-report questionnaire rather than interview (the latter might lead to under-reporting), number of binge episodes and levels of background drinking. In particular, our definition of binge-pattern drinking may have under-estimated risk when compared with studies using binge drinking definitions that reflect alcohol consumption over a shorter duration or studies assessing the impact of frequent binge episodes [37]. However, our findings highlight potential risk to the offspring through exposure to a minimum of 32 g of alcohol. Finally, other study limitations include the possibility of unmeasured or residual confounding (for example, we did not have information on family history of ADHD), the mental health outcome measures were based on a brief questionnaire (SDQ), sample attrition involving missing data on the exposure at 32 weeks gestation and response rates for the outcome measures, the influence of multiple testing as outcomes were assessed using information from different sources, and the representativeness of responders to the parent-completed SDQ. However, there was less bias in the availability of teacher or KS2 data and there is evidence that differential attrition within ALSPAC does not affect estimations of risk of behavioural disorder [38].

Our findings suggest a persistent adverse effect over time for children’s mental health related to prenatal exposure to ≥4 drinks in a single day. This pattern of drinking on one or more days during pregnancy carries risk in the absence of daily drinking. The pattern of findings at age 11 years was similar to our earlier findings following up this cohort up till age 7. The persistence of adverse effects into mid-childhood, even with small individual effect sizes, is significant at a whole-population level. Clinicians should enquire about episodic binge drinking as well as regular drinking when taking a history of pregnancy and keep this in mind when assessing mental health and learning problems [15, 18, 27, 28]. At an individual level, pregnant women should be aware of possible risks associated with episodic binge-pattern drinking, even if this occurs on an occasional basis. Our findings have implications for clear policy messages about patterns of alcohol consumption during pregnancy, whereby women who choose to drink occasionally should avoid having several drinks in a day.

## Electronic supplementary material

Below is the link to the electronic supplementary material.

Supplementary material 1 (DOC 22 kb)

Supplementary material 2 (DOC 95 kb)
