# Supplementary material for: Prenatal exposure to binge pattern of alcohol consumption: mental health and learning outcomes at age 11
Source: Eur Child Adolesc Psychiatry. 2014 Sep 11;23(10):891–9. doi: 10.1007/s00787-014-0599-7 (PMC4186965; doi:10.1007/s00787-014-0599-7)
Supplement: Supplementary file 1 — Supplementary material 1 (DOC 22 kb) [file 787_2014_599_MOESM1_ESM.doc]

**Online Supplementary Figure**

**TEACHER SDQs**

**(n = 4274)**

**KS2 EXAM RESULTS**

**(n = 6939)**

**ELIGIBLE SAMPLE**

**(n = 13,171)**

**18-WEEK DATA**

**(n = 12,257)**

**32-WEEK QUESTIONS NOT ASKED**

**(n = 3378)**

**32-WEEK QUESTIONS NON-RESPONSE**

**(n = 914)**

**SAMPLE FOR ANALYSIS**

**(18 & 32 WEEK DATA)**

**(n = 7965)**

**PARENT SDQs**

**(n = 4610)**
