# Supplementary material for: Prenatal exposure to binge pattern of alcohol consumption: mental health and learning outcomes at age 11
Source: Eur Child Adolesc Psychiatry. 2014 Sep 11;23(10):891–9. doi: 10.1007/s00787-014-0599-7 (PMC4186965; doi:10.1007/s00787-014-0599-7)
Supplement: Supplementary file 2 — Supplementary material 2 (DOC 95 kb) [file 787_2014_599_MOESM2_ESM.doc]

**Online Supplementary Table 1: Parent SDQ Availability**

|  | **Data available**  n = 4610 | **Missing data**  n = 3355 | **χ^2^**  **(1 d.f.)** | **p value** |
| --- | --- | --- | --- | --- |
| **Prenatal Factors** |  |  |  |  |
| Maternal age (≥35 years) | 12% | 8% | 28.44 | <0.001 |
| Any smoking | 13% | 26% | 209.15 | <0.001 |
| Cannabis use | 2% | 2% | 7.14 | 0.008 |
| Illicit drug use | 0.4% | 0.5% | 0.05 | 0.820 |
| Parity (≥1) | 54% | 58% | 10.27 | 0.001 |
| Highest maternal education (‘O’ level or above) | 79% | 59% | 374.50 | <0.001 |
| Own home | 84% | 66% | 345.34 | <0.001 |
| Currently married | 83% | 72% | 147.73 | <0.001 |
| Maternal depression | 10% | 16% | 58.06 | <0.001 |
| ≥4 drinks at 14-18 weeks | 22% | 28% | 40.08 | <0.001 |
| **Child Factors** |  |  |  |  |
| Gestational age (≤36 weeks) | 4% | 5% | 1.18 | 0.277 |
| Ethnicity (non-white) | 2% | 3% | 4.01 | 0.045 |
| Gender (male) | 51% | 52% | 2.06 | 0.151 |
| Birth weight (kg) | 3.45 (0.52) | 3.43 (0.53) | t=2.14 | 0.032 |

% or mean (s.d.)

**Online Supplementary Table 2: Teacher SDQ Availability**

|  | **Data available**  n = 4274 | **Missing data**  n = 3691 | **χ^2^**  **(1 d.f.)** | **p value** |
| --- | --- | --- | --- | --- |
| **Prenatal Factors** |  |  |  |  |
| Maternal age (≥35 years) | 10% | 11% | 1.15 | 0.284 |
| Any smoking | 19% | 18% | 0.97 | 0.324 |
| Cannabis use | 1% | 2% | 9.45 | 0.002 |
| Illicit drug use | 0.4% | 0.5% | 0.62 | 0.433 |
| Parity (≥1) | 56% | 56% | 0.00 | 1.000 |
| Highest maternal education (‘O’ level or above) | 70% | 72% | 1.70 | 0.192 |
| Own home | 78% | 75% | 13.94 | <0.001 |
| Currently married | 79% | 77% | 5.56 | 0.018 |
| Maternal depression | 13% | 12% | 1.27 | 0.261 |
| ≥4 drinks at 14-18 weeks | 24% | 24% | 0.19 | 0.663 |
| **Child Factors** |  |  |  |  |
| Gestational age (≤36 weeks) | 5% | 4% | 0.28 | 0.594 |
| Ethnicity (non-white) | 2% | 3% | 3.05 | 0.081 |
| Gender (male) | 51% | 52% | 0.72 | 0.396 |
| Birth weight (kg) | 3.44 (0.53) | 3.45 (0.52) | t=0.31 | 0.759 |

% or mean (s.d.)

**Online Supplementary Table 3: Key Stage 2 data Availability**

|  | **Data available**  n = 6939 | **Missing data**  n = 1026 | **χ^2^**  **(1 d.f.)** | **p value** |
| --- | --- | --- | --- | --- |
| **Prenatal Factors** |  |  |  |  |
| Maternal age (≥35 years) | 10% | 12% | 2.93 | 0.087 |
| Any smoking | 19% | 19% | 0.02 | 0.704 |
| Cannabis use | 2% | 3% | 8.23 | 0.004 |
| Illicit drug use | 0.4% | 0.4% | 0.00 | 1.000 |
| Parity (≥1) | 56% | 52% | 6.47 | 0.011 |
| Highest maternal education (‘O’ level or above) | 70% | 76% | 13.06 | <0.001 |
| Own home | 78% | 71% | 25.11 | <0.001 |
| Currently married | 79% | 74% | 10.38 | 0.001 |
| Maternal depression | 12% | 14% | 0.92 | 0.337 |
| ≥4 drinks at 14-18 weeks | 24% | 24% | 0.00 | 1.000 |
| **Child Factors** |  |  |  |  |
| Gestational age (≤36 weeks) | 4% | 5% | 0.10 | 0.751 |
| Ethnicity (non-white) | 2% | 3% | 0.90 | 0.343 |
| Gender (male) | 51% | 54% | 3.78 | 0.052 |
| Birth weight (kg) | 3.44 (0.52) | 3.43 (0.54) | t=0.42 | 0.677 |

% or mean (s.d.)
